# Feasibility of a Novel COVID-19 Telehealth Care Management Program Among Individuals Receiving Treatment for Opioid Use Disorder: Analysis of a Pilot Program

**DOI:** 10.2196/39772

**Published:** 2022-08-30

**Authors:** Kimberly D Williams, Claudine T Jurkovitz, Mia A Papas, Ann Kathryn Muther, Sharon L Anderson, Tammy L Anderson

**Affiliations:** 1 Institute for Research on Equity and Community Health ChristianaCare Wilmington, DE United States; 2 Center for Virtual Health ChristianaCare Wilmington, DE United States; 3 Department of Sociology and Criminal Justice University of Delaware Newark, DE United States

**Keywords:** opioid use disorder, substance use, drug addiction, opioid treatment program, COVID-19, telehealth, telemedicine, eHealth, Short Message Service, SMS, text messaging, text message, opioid use, opioid, care management, patient care management, health intervention, telehealth intervention

## Abstract

**Background:**

The emergence of COVID-19 exacerbated the existing epidemic of opioid use disorder (OUD) across the United States due to the disruption of in-person treatment and support services. Increased use of technology including telehealth and the development of new partnerships may facilitate coordinated treatment interventions that comprehensively address the health and well-being of individuals with OUD.

**Objective:**

The analysis of this pilot program aimed to determine the feasibility of delivering a COVID-19 telehealth care management program using SMS text messages for patients receiving OUD treatment.

**Methods:**

Eligible individuals were identified from a statewide opioid treatment program (OTP) network. Those who screened positive for COVID-19 symptoms were invited to connect to care management through a secure SMS text message that was compliant with Health Insurance Portability and Accountability Act standards. Care management monitoring for COVID-19 was provided for a period of up to 14 days. Monitoring services consisted of daily SMS text messages from the care manager inquiring about the participant’s physical health in relation to COVID-19 symptoms by confirming their temperature, if the participant was feeling worse since the prior day, and if the participant was experiencing symptoms such as coughing or shortness of breath. If COVID-19 symptoms worsened during this observation period, the care manager was instructed to refer participants to the hospital for acute care services. The feasibility of the telehealth care management intervention was assessed by the rates of adoption in terms of program enrollment, engagement as measured by the number of SMS text message responses per participant, and retention in terms of the number of days participants remained in the program.

**Results:**

Between January and April 2021, OTP staff members referred 21 patients with COVID-19 symptoms, and 18 (82%) agreed to be contacted by a care manager. Participants ranged in age from 27 to 65 years and primarily identified as female (n=12, 67%) and White (n=15, 83%). The majority of participants were Medicaid recipients (n=14, 78%). There were no statistically significant differences in the demographic characteristics between those enrolled and not enrolled in the program. A total of 12 (67%) patients were enrolled in the program, with 2 (11%) opting out of SMS text message communication and choosing instead to speak with a care manager verbally by telephone. The remaining 10 participants answered a median of 7 (IQR 4-10) SMS text messages and were enrolled in the program for a median of 9 (IQR 7.5-12) days. No participants were referred for acute care services or hospitalized during program enrollment.

**Conclusions:**

These results demonstrate the feasibility of a novel telehealth intervention to monitor COVID-19 symptoms among OTP patients in treatment for OUD. Further research is needed to determine the applicability of this intervention to monitor patients with comorbid chronic conditions in addition to the acceptability among patients and providers using the SMS text messaging modality.

## Introduction

When the COVID-19 *pandemic* began in the United States, it collided with a multidecade opioid *epidemic*. Following the emergence of the pandemic in March 2020, drug overdoses spiked by 28.5% between April 2020 and April 2021 [1]. Provisional data reported that approximately 75% of drug overdose deaths in 2020 were due to opioids including potent synthetic opioids such as fentanyl [2].

As overdose deaths continue to increase, improving access to evidence-based treatment for opioid use disorder (OUD) is a key tertiary prevention strategy [3]. This necessitates coordinating efforts with treatment settings including opioid treatment programs (OTPs). However, patients face challenges in meeting recovery and other health goals while adhering to COVID-19 safety precautions. Specifically, calls for increased social distancing to reduce disease spread may impede access to OUD treatment services [4]. Thus, there are concerns that the COVID-19 pandemic may exacerbate the current opioid crisis [5]. People with OUD may also be at higher risk for contracting COVID-19 and experiencing more severe outcomes in terms of mortality and morbidity [4,6-9].

To address these concerns, experts recommend increased use of technology, including telehealth, and the development of new partnerships to facilitate coordinated treatment interventions that comprehensively address individual health and well-being [10,11]. While this could help manage the needs associated with co-occurring OUD and COVID-19, there is limited evidence on how to best engage and retain patients with OUD in telehealth care management services. This retrospective study aimed to determine the feasibility of a COVID-19 telehealth care management program using a standardized protocol of SMS text messaging for patients receiving OUD treatment in a statewide OTP network.

## Methods

### Pilot Program Setting and Participants

An OTP network based in the state of Delaware recruited patients to participate in this telehealth care management program where eligible patients are invited to connect with a care manager to monitor their COVID-19 symptoms through daily SMS text messaging communications. The overall COVID-19 telehealth care management program has been in place since the beginning of the pandemic and provides COVID-19 resources and support to businesses and their employees. It consists of 10 care managers who are all licensed registered nurses. This study focuses on the feasibility of enrolling patients with OUD into this established telehealth program.

OTP staff conducted universal COVID-19 symptom screenings for their patients during OTP appointments and referred patients with symptoms to community sites for a COVID-19 test to confirm diagnosis. The Delaware Department of Public Health reviewed and verified diagnostic test samples, and notified the testing facility if patients were positive for COVID-19.

Eligible patients who screened positive for COVID-19 symptoms were invited by OTP staff to participate in the telehealth care management program. Eligibility criteria included patients 18 years or older; with confirmed COVID-19 symptoms; currently receiving OUD treatment; who owned a cell phone with SMS text messaging capabilities; and able to understand, speak, and read English.

### Program Procedures

Beginning January 2021, OTP staff invited patients who met eligibility criteria to receive a SMS text message from a care manager using Twistle software [12]. This enabled a 2-way communication between the care managers and program participants that was compliant with Health Insurance Portability and Accountability Act standards. Program monitoring services consist of a standardized protocol for daily SMS text messages sent from care managers to patient participants inquiring about the following: the participant’s physical health status in relation to COVID-19 symptoms by confirming their current temperature, if the participant is feeling worse since the prior day, and if the participant is experiencing symptoms such as coughing or shortness of breath. Individuals with OUD participating in the pilot program were monitored up to 14 days while continuing OUD treatment at the OTP. The length of the monitoring period during this pilot program was based on the Centers for Disease Control and Prevention (CDC) COVID-19 quarantine and isolation guidelines that were in place for the general population as of January 2021. These guidelines were later updated by the CDC in December 2021 [13]. If any participants reported worsening COVID-19 symptoms during this monitoring period, the care manager would conduct a standardized assessment to obtain additional details about their symptoms. This assessment included a brief survey asking participants to select their current temperature in terms of Fahrenheit degree categories: 103 °F (39.4 °C) or greater, 101.6 °F to 102.9 °F (38.7 °C to 39.4 °C), 100.4 °F to 101.5 °F (38 °C to 38.6 °C), 97 °F to 100.3 °F (36.1 °C to 37.9 °C), or less than 97 °F (36.1 °C). Participants were also asked to confirm if they were experiencing any coughing or shortness of breath. Care managers would use this clinical information to help determine when patients needed a referral to a local health care provider for acute care services or hospitalization.

### Data Sources and Measures

This pilot program was conducted between January 11 and April 30, 2021. We assessed the feasibility of the program intervention by focusing on three process measures: (1) adoption, which was measured by the number of patients who were invited versus the number who agreed to participate and enrolled in the program; (2) engagement, as measured by the overall number of SMS text messages sent by participants; and (3) program retention, as measured by the number of days participants remained active in the program by continuing to respond to the daily SMS text messages. OTP staff provided a count of patients screened for COVID-19 symptoms and program referrals. The lead care manager tallied the number of patients who screened positive for COVID-19, received a SMS text message program invitation, were lost to follow-up or declined participation, and were enrolled in the program. They also reported the number of days participants were monitored and if any were referred for acute care services during the program. The Twistle data provided the number of messages sent by participants to care managers. Demographic data including age, sex, race, and insurance type were obtained from the electronic health record (EHR). An EHR review confirmed if participants were locally hospitalized while in the program.

### Analysis

Descriptive statistics summarized the characteristics of patients approached to participate in the telehealth care management pilot program and process metrics for program enrollment. Mann-Whitney *U* and Fisher exact tests calculated differences in the demographic characteristics for age, sex, race, and insurance type between those enrolled and not enrolled in the program. Data were analyzed using Stata/SE version 16.1 (StataCorp).

### Ethics Approval

We received approval from the ChristianaCare Institutional Review Board (IRB00000480) to evaluate the feasibility of this program.

## Results

**Figure 1:**
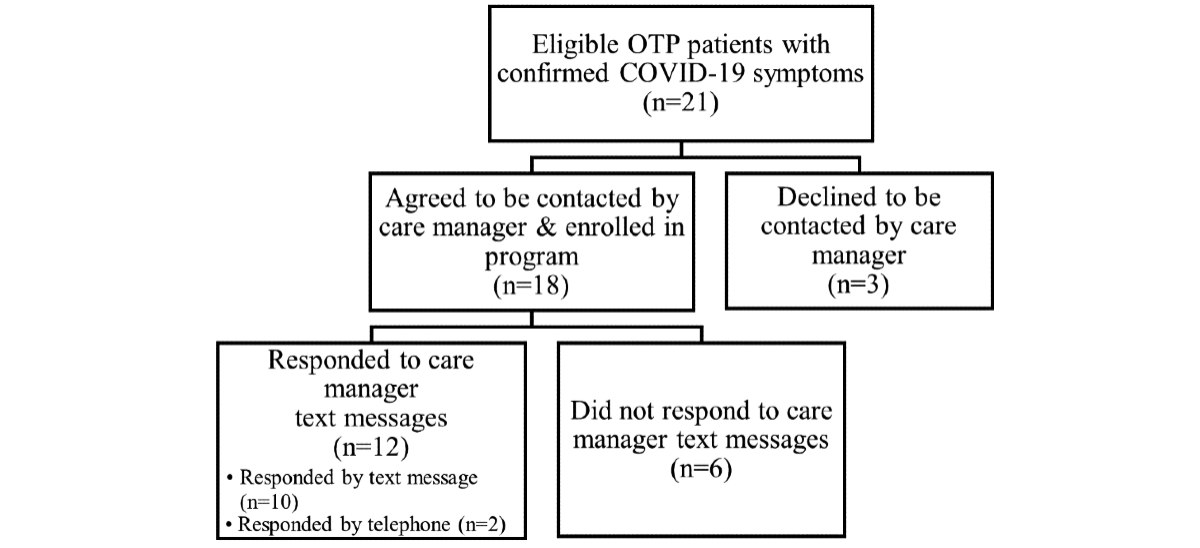
Flow diagram of OTP participants in the COVID-19 telehealth care management program. OTP: opioid treatment program.

Figure 1 provides a flow diagram of the participation of OTP patients in the COVID-19 telehealth care management program. Of the 21 eligible patients with COVID-19 symptoms, 18 (82%) agreed to be contacted by a care manager. Of those, 6 (33%) did not enroll in the program, including 4 not responding to care manager messages, 1 opting out, and 1 being withdrawn after confirming a negative COVID-19 diagnostic test. This left 12 (67%) individuals who participated in the telehealth care management program.

Characteristics of patients enrolled in the program (n=12) and those not enrolled (n=6) are shown in Table 1. Overall, participants ranged in age from 27 to 65 years and primarily identified as female (n=12, 67%) and White (n=15, 83%). The majority of participants were Medicaid recipients (n=14, 78%) with the remaining participants enrolled in either a Medicare, private, or dual Medicaid/Medicare plan (n=3, 17%). There were no statistically significant differences in demographic characteristics between those enrolled and not enrolled in the program (Table 1).

Of the 12 individuals enrolled, 2 opted-out of SMS text message communication and instead spoke with the care manager verbally by telephone. The remaining 10 participants answered a median of 7 (IQR 4-10) messages. The median time in the program was 9 (IQR 7.5-12) days. Of the 12 participants, 10 were monitored for less than 14 days because of improvement in COVID-19 symptoms, and 2 were monitored for the maximum 14-day period. No participants were referred for acute care services or hospitalized while being monitored by the care manager.

**Table 1:** Characteristics of opioid treatment program patients enrolled and not enrolled in the COVID-19 telehealth care management program.

		All patients (N=18)	Enrolled (n=12)	Not enrolled^a^ (n=6)	*P* value	
Age (years), mean (SD)		41.47 (11.43)^b^	43.0 (11.60)	37.80 (11.34)^b^	.41	
**Sex, n (%)**						.10
	Female	12 (67)	7 (58)	5 (83)		
	Male	5 (28)	5 (42)	0 (0)		
	Unknown, not reported	1 (6)	0 (0)	1 (17)		
**Race, n (%)**						.25
	White	15 (83)	11 (92)	4 (67)		
	Other^c^	2 (11)	1 (8)	1 (17)		
	Unknown, not reported	1 (6)	0 (0)	1 (17)		
**Insurance type, n (%)**						.19
	Medicaid	14 (78)	9 (75)	5 (83)		
	Other^d^	3 (17)	3 (25)	0 (0)		
	Unknown, not reported	1 (6)	0 (0)	1 (17)		

^a^Patients that were not enrolled either declined participation or were lost to follow-up after agreeing to be contacted by a telehealth care manager.

^b^Age for 1 participant not reported.

^c^Other race: due to the small sample size, participants with race identified as “Black/African American” or “Other” were combined into one category.

^d^Other insurance type: due to the small sample size, participants with insurance type identified as “Medicare,” “Private” plan, or “dual Medicaid/Medicare” plan were combined into one category.

## Discussion

### Principal Results

In this study, we assessed the feasibility of using SMS text messaging to manage COVID-19 symptoms of OTP patients receiving treatment for OUD. We evaluated the feasibility using three criteria: adoption measured by the ratio of enrolled participants over eligible patients, engagement measured by the number of participant responses to the care managers, and retention measured by the days participants remained in the program. Although a small number of individuals were eligible, the majority of those approached about the program agreed to be contacted and monitored by a care manager via SMS text message. Only a few then opted for verbal telephone communication as opposed to SMS text message communication. Our findings are consistent with the literature documenting the acceptability of SMS text message interventions among individuals seeking OUD treatment following emergency department discharge [14]. Adherence with our SMS text message protocol was high, with the majority of participants responding multiple times to the care manager messages during the monitoring period.

Telehealth is considered safe and effective for a variety of conditions, and research has demonstrated its ability to manage health care needs while maintaining social distancing to reduce infectious disease transmission [15-17]. Current research involving individuals with substance use disorders (SUDs), including OUD, primarily address the provision of telehealth services for SUD treatment including medication management and counseling [18-20]. Little is known about telehealth’s ability to manage co-occurring medical concerns while receiving SUD treatment. The convenience of offering telehealth-based care management for COVID-19 could ease the burden of managing multiple conditions and potentially improve retention in SUD treatment programs.

Telehealth care management through secure SMS text messaging is a promising tool that offers a convenient, private, and low-maintenance approach to promote patient engagement. SMS text messaging does not require broadband internet access, a smartphone data plan, or downloading a smartphone app, thereby avoiding barriers that exist for many individuals including those in rural areas [21]. Though care management delivered through SMS text messaging is acknowledged to be cost-effective and a relatively simple telehealth modality, few studies have explored its application among individuals receiving treatment for SUDs including OUD [20].

Our study shows that OTP patients may be comfortable communicating through SMS text messages. Though 2 participants preferred to communicate verbally by phone, we believe SMS text messaging remains a feasible telehealth modality. Further research is needed to explore telehealth preferences including concerns related to privacy, security, and other potential barriers. This is consistent with a recent review identifying that only a limited number of studies to date have explored telehealth *acceptability* among patients and providers [22]. Consequently, more participatory research on telehealth use is necessary to better address patient engagement needs and ensure sustained telehealth use among patients and providers [22,23].

### Limitations

The small sample size of this pilot study limits the generalizability of our findings to the broader population of OTP patients. Because of the small sample size, we could not assess other feasibility criteria such as estimating the appropriate workload for the care manager or the referral rate to acute care. The program focused on patients from a single OTP network within Delaware, which restricts the validity of our findings to this local region. The feasibility of this program may vary depending on contextual factors such as geography. Fortunately, our OTP network included multiple community-based sites in urban, suburban, and rural settings throughout the state.

### Conclusions

This study identified the feasibility of a novel telehealth program intervention delivering COVID-19 care management services for OTP patients. Further research is needed to determine the impact of this intervention on managing other comorbid chronic conditions within this patient population, the association between telehealth outcomes and use of health care services, and telehealth acceptability among patients treated for OUD and providers to ensure engagement and sustainability of services through this evolving modality using SMS text messaging for telehealth purposes.

## Abbreviations

CDC: Centers for Disease Control and Prevention
EHR: electronic health record
OTP: opioid treatment program
OUD: opioid use disorder
SUD: substance use disorder
